# Giant left atrial myxoma causing acute ischemic stroke in a child

**DOI:** 10.1186/s40792-017-0411-2

**Published:** 2018-01-12

**Authors:** Hayato Ise, Natsuya Ishikawa, Sentaro Nakanishi, Hiroyuki Kamiya

**Affiliations:** Department of Cardiac Surgery, Asahikawa Medical University, Midorigaoka-Higashi 2-1-1-1, Asahikawa, Hokkaido 078-8510 Japan

**Keywords:** Myxoma, Ischemic stroke, Hemiparesis, Pediatric

## Abstract

Ischemic stroke is uncommon in pediatric populations and is sometimes caused by cardiac myxoma. In such cases, neurological deficits initially present in ischemic stroke due to emboli or thrombi of the myxoma. Echocardiography is helpful to diagnose myxoma in a timely manner and allows urgent surgical resection of the myxoma. We report a successful case of myxoma in a 7-year-old boy who initially presented with left-sided hemiparesis.

## Background

Ischemic stroke is uncommon in pediatric populations, particularly that caused by primary cardiac tumor. We report a rare case of a 7-year-old boy who presented with left-sided hemiparesis caused by ischemic stroke due to left atrial myxoma.

## Case presentation

**Fig. 1:**
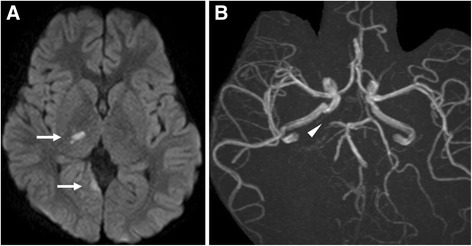
**a** Magnetic resonance imaging shows ischemic strokes in the right thalamus and right occipital lobe (arrows). **b** Magnetic resonance angiography shows obstruction of the right posterior cerebral artery (arrowhead)

**Fig. 2:**
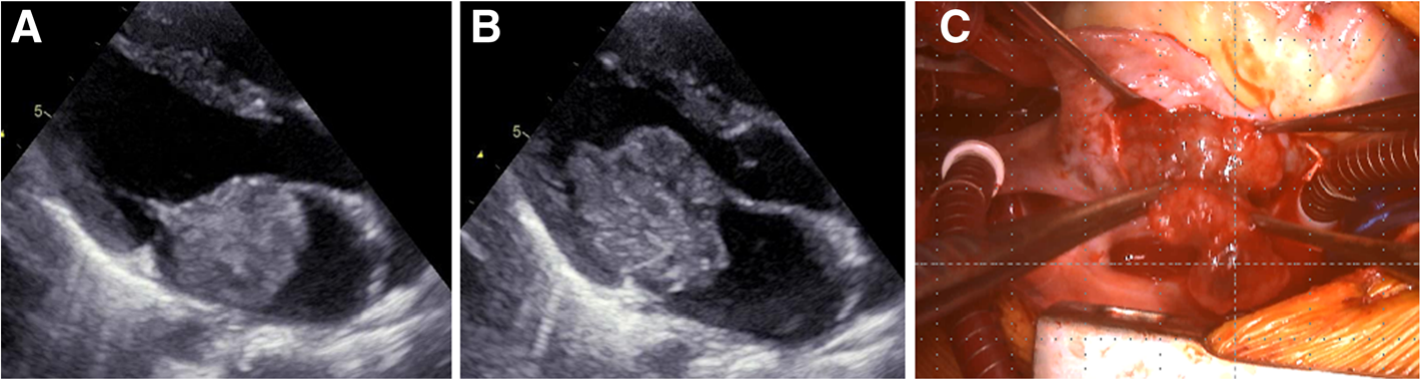
Echocardiography shows a giant mass in the left atrium (**a**), which prolapsed into the left ventricle during diastole (**b**). **c** A giant gelatinous tumor in the left atrium

A 7-year-old boy who presented with dizziness, headache, and left-sided hemiparesis was transported by ambulance to the pediatric department of another hospital. He was admitted for further investigations. He had been born with developmental disorders. From 1 year before this admission, he had complained of dizziness and nausea once a week but had been diagnosed with orthostatic dysregulation at another hospital at that time. After admission, he underwent further investigations. Magnetic resonance imaging (MRI) revealed ischemic strokes in the right thalamus, right occipital lobe, and bilateral frontal lobes (Fig. 1a). Magnetic resonance angiography (MRA) demonstrated obstruction of the right posterior cerebral artery (Fig. 1b). Echocardiography detected a 4 × 3-cm giant mass that with unclear origin in the left atrium (Fig. 2a). This mass was prolapsing into the left ventricle during diastole (Fig. 2b). The left atrium was dilated, and moderate mitral valve regurgitation was noted. He was diagnosed with an ischemic stroke due to embolism caused by cardiac tumor. He was then transported to our hospital, where we performed emergent surgical resection of the tumor. Under a median sternotomy approach, cardiopulmonary bypass was instituted by aortic and bicaval cannulation. After inducing cardioplegic arrest, the right atrium was opened. The atrial septum was incised at the fossa ovalis, and a giant gelatinous tumor inside the left atrium appeared (Fig. 2c). The neck of the tumor was adherent to the left atrial side of the atrial septum. The tumor and its neck, including part of the atrial septum, were fully resected and the atrial septum was directly closed. The patient had no difficulty with weaning from cardiopulmonary bypass and was extubated 3 h after surgery. Histopathological diagnosis was myxoma. Postoperative echocardiography showed no residual tumor, and only mild mitral valve regurgitation was noted. Echocardiography results from his sister and parents which were collected at his admission were normal. He was discharged to his home on postoperative day 19, by which time he had almost recovered from left-sided hemiparesis and could walk unaided.

## Discussion and conclusions

Ischemic stroke in children is extremely rare, with a reported incidence of 2–13 cases per 100,000 children [1]. This clinical condition increases long-term morbidity (50–80%) and mortality (up to 20%) [2]. In children, ischemic stroke can be caused by conditions such as tumor, endocarditis, vasculopathy, thrombotic disorders, and inflammatory disorders [3, 4]. Ischemic stroke due to myxoma in children younger than 18 years old is extremely rare. Only 17 cases have been reported in the literature [2].

Also, primary cardiac tumor is uncommon in pediatric populations. Myxoma is the most common in adults but is the third most next to rhabdomyoma and fibroma in children [3]. Because the myxomas are gelatinous and fragile, these sometimes cause ischemic stroke in the children also. Myxoma in children reveals various symptoms depending on the size and location, and therefore, it is difficult to diagnose appropriately.

Many of the patients with myxoma present with one or more symptoms of Goodwin’s triad, which include embolism, intracardiac obstruction, and constitutional symptoms [5]. Embolism is the most common symptom in atrial myxoma, which occurs in 20–45% of myxomas, so neurological deficits due to embolic stroke are the first symptoms of myxoma in some cases [2, 3]. Peripheral cutaneous embolic phenomena have also been reported as “red spots” or “rash,” which often presents before cerebral ischemic events [2, 3]. In children, particularly infants, intracardiac obstruction is more common than stroke, because of the small heart cavity. Congestive heart failure was seen in 64% of infant patients [2]. Constitutional symptoms in myxoma patients typically include fever, weight loss, fatigue, and dizziness.

In our case, the patient presented with dizziness and nausea once a week from 1 year before admission. These symptoms might have represented constitutional symptoms of myxoma and some emboli or thrombi of myxoma causing minor strokes in the brain. If echocardiography had been performed at that time, cardiac myxoma might have been detected earlier at a smaller size. On this admission, echocardiography was immediately performed after he presented with neurological deficit. This allowed us to perform emergent tumor resection and prevented occurring additional embolism due to myxoma.

Cardiac myxoma in children is uncommon but sometimes causes ischemic stroke and further complications due to thrombi or emboli. Emergent resection of the tumor is the best treatment at present for children and thus warrants immediate and precise diagnosis. Our experience suggests that echocardiography should be performed when children present with unusual neurological deficits.

## References

[1] Lynch JK, Hirtz DG, DeVeber G, Nelson KB (2002). Report of the National Institute of Neurological Disorders and Stroke workshop on perinatal and childhood stroke. Pediatrics.

[2] Jenifer F, David L, Don M (2014). Cardiac myxoma causing acute ischemic stroke in a pediatric patient and a review of literature. Pediatr Neurol.

[3] Al-Mateen M, Hood M, Trippel D, Samuel JI, Otto RK, Vitikanen KJ (2003). Cerebral embolism from atrial myxoma in pediatric patients. Pediatrics.

[4] van den Wijngaard I, Wermer M, van Walderveen MA, Wiendels N, Peeters-Scholte C, Lycklama GJ (2014). Intra-arterial treatment in a child with embolic stroke due to atrial myxoma. Interv Neuroradiol.

[5] Sernich S, Chauhan A, Singh D, Fuchs H, Caspi J (2012). Left atrial myxoma in a child: a challenging diagnosis of rare lesion. World Journal for Pediatric and Congenital Heart Surgery.

